# The fixed cortical fraction assumption in Hip Structure Analysis produces systematic, sex- and age- dependent errors in femoral neck morphology estimates

**DOI:** 10.3389/fendo.2026.1932381

**Published:** 2026-10-08

**Authors:** Madison S. Richards, Erin M. R. Bigelow, Robert W. Goulet, Bonnie T. Brooker-Nolan, Kyle J. Franklin, Karl J. Jepsen, Todd L. Bredbenner

**Affiliations:** 1 Department of Orthopaedic Surgery, University of Michigan, Ann Arbor, MI, United States; 2 Department of Mechanical and Aerospace Engineering, University of Colorado Colorado Springs, Colorado Springs, CO, United States

**Keywords:** bone mineral density (BMD), computed tomography (CT), cortical fraction, dual-energy x-ray absorptiometry (DXA), femoral neck geometry, fracture risk, hip structure analysis/HSA, osteoporosis

## Abstract

Hip Structure Analysis (HSA) estimates femoral neck morphology from dual-energy X-ray absorptiometry (DXA) images using simplifying assumptions, including that cortical bone accounts for a fixed 60% of total bone mineral content at the narrow neck (cortical fraction, *f_c_* = 0.6). The empirical basis of this assumption is poorly established, and its effect on HSA-derived structural parameters has not been systematically evaluated. PseudoDXA images were generated by direct projection of nanoCT volumes from cadaveric proximal femurs from 32 White female (ages 24–89+ years) and 30 White male (ages 18–89 years) donors. Cortical and trabecular bone were segmented separately, enabling direct measurement of *f_c_* and comparison of HSA estimates using fixed versus measured *f_c_* against nanoCT-derived reference values. Measured *f_c_* ranged from 0.23 to 0.67 and averaged 0.48 for females and 0.42 for males, both substantially below 0.6. In females, *f_c_* increased significantly with age; in males, *f_c_* decreased significantly with body weight. Using fixed *f_c_* produced large, systematic errors in HSA-derived cortical area and buckling ratio that were sex- and age-specific, reaching up to 36% for cortical area in younger individuals. Replacing fixed *f_c_* with measured *f_c_* significantly reduced errors for cortical area in both sexes and for buckling ratio in males, with significantly greater improvement in concordance for cortical area and inner radius in males. Buckling ratio errors remained large in females even with measured *f_c_*, consistent with an additional departure from the uniform cortical thickness assumption, which is hypothesized to be more pronounced in females due to the disproportionately thick inferior cortex. These findings demonstrate that the fixed *f_c_* assumption introduces systematic, sex- and age-specific errors into HSA-derived structural estimates, and that development of DXA-derived individualized *f_c_* estimation represents a meaningful step toward improving the clinical reliability of HSA for fracture risk assessment.

## Introduction

Osteoporotic hip fractures were estimated at 14.2 million globally in 2019, with annual increases in the age-standardized rate (1, 2), resulting in 2.9 million years lived with disability (2) and increased mortality rates, particularly for males and older individuals (3). Monitoring hip strength is critical for identifying individuals at increased fracture risk who may benefit from preventive treatment. Because hip strength cannot be measured directly in living humans, bone health assessments must rely on estimates of bone mass and structure derived from noninvasive imaging technologies as surrogate measures of strength (4–6). Age-related declines in hip strength arise in part from an imbalance between bone resorption and formation leading to increased porosity, cortical thinning, and loss of trabecular bone volume fraction (BV/TV). Recent work reported that age-related changes in femoral neck structure vary significantly among individuals depending on their external bone size (7). We propose that assessments of bone strength and strength decline should account for structural heterogeneity across the population and variation in biomechanical pathways increasing fracture risk. Hip Structure Analysis (HSA) is widely used to estimate femoral neck (FN) structure from hip DXA images (**Figure 1**); however, it is unclear whether the simplifying assumptions used in HSA introduce systematic errors that could lead to bias (8–10), particularly for parameters that depend on assumptions about cortical bone distribution (**Figure 1**).

**Figure 1:**
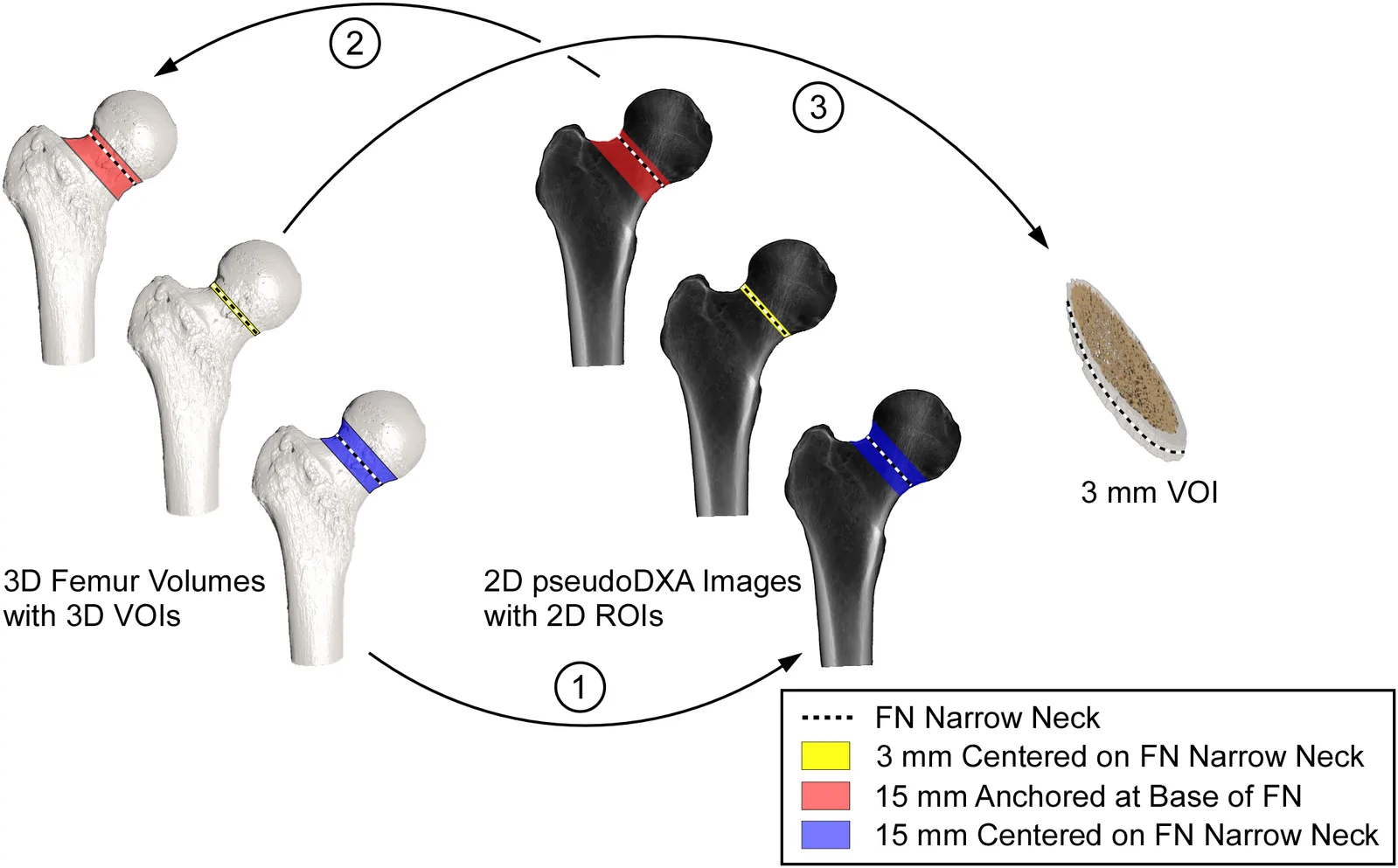
Schematic illustrating the pseudoDXA image generation and femoral neck (FN) VOI extraction workflow. (1) Three-dimensional nanoCT femur volumes were projected onto a plane with the proximal femur anteverted 15 degrees to generate 2D pseudoDXA images. Three femoral neck ROIs were positioned on each pseudoDXA image corresponding to the narrow neck location (dashed white line): a 3 mm VOI centered on the FN narrow neck (yellow), a 15 mm VOI centered on the FN narrow neck (blue), and a 15 mm VOI anchored at the base of the FN (red). (2) The 2D ROI boundaries were back-projected onto the 3D nanoCT femur volumes to define the corresponding 3D VOIs. (3) The 3D VOIs were extracted for subsequent cortical and trabecular segmentation and computation of ground truth morphological parameters; the 3 mm narrow neck VOI (shown) coincides with the region used by the HSA algorithm.

HSA has been widely used to assess hip structural changes associated with aging (11, 12) kidney transplantation (13), and type 1 diabetes (14), demonstrating its value for characterizing structural aspects of bone strength in diverse clinical applications. HSA estimates FN structural measures including bone width, cross-sectional area (*CSA*), cross-sectional moment of inertia (*CSMI*), and section modulus (*Z*) by integrating mineral X-ray attenuation profiles (11, 15, 16). CSA reflects the total load-bearing area of bone at a given cross-section, CSMI quantifies resistance to bending about the neutral axis, and *Z = CSMI/c* provides an estimate of bending strength (where *c* is the distance from the neutral axis to the outer surface). These structural measures correlate well with those measured from corresponding regions of computed tomography images (9). HSA also estimates *R_inner_* (*endocortical diameter/2*), buckling ratio (*BR*), and cortical area (*Ct.Ar*) using a suite of algorithms along with simplifying assumptions (11, 15, 16). For these parameters, HSA assumes the femoral neck has a circular cross-sectional shape with a uniformly thick cortical shell that accounts for 60% of the femoral neck bone mineral content (BMC). Accurate estimation of these parameters is necessary because BR and Ct.Ar directly indicate cortical instability and load-bearing capacity, respectively, and estimation errors could lead to mischaracterizing fracture risk. The extent to which these assumptions are associated with systematic errors when estimating *R_inner_*, *Ct.Ar*, and *BR* has not been evaluated.

The ratio of cortical to total mineral content, designated as *f_c_* in the HSA algorithms, is assumed to be 0.6 at the narrow neck regardless of factors such as sex, race/ethnicity, age, or disease condition (11). However, we reported that the proportion of cortical to total bone mineral content is not fixed but varies with sex (17). Given known age-related changes in bone structure (7), *f_c_* would also be expected to vary with age. The goals of the current study were three-fold: first, to assess how the ratio of cortical to total mineral content (*f_c_*) varies with sex, age, and body size characteristics using high-resolution nano-computed tomography (nanoCT) as the reference standard; second, to assess the errors associated with using a fixed *f_c_* (*f_c_* = 0.6) when estimating femoral neck morphology compared to structural measures determined from nanoCT data; and third, to determine if using the measured *f_c_* would reduce the errors associated with HSA estimates compared to using a fixed *f_c_*.

## Methods

### Human tissue samples

Cadaveric femora from 32 White female and 30 White male donors were obtained from Science Care (Phoenix, AZ, USA), Anatomy Gifts Registry (Hanover, MD, USA), and the University of Michigan Anatomical Donations program (Ann Arbor, MI, USA). Samples were excluded if they had known or suspected musculoskeletal pathologies. Human tissue use and handling were approved by the Institutional Biosafety Committee (IBC), and the study was exempted by the Institutional Review Board (IRB). All samples were cleaned of soft tissue and prepared for high-resolution imaging by sectioning the femoral shaft at a location 16.5 cm distal to the superior aspect of the femoral head. The proximal femur samples were embedded in a 5 cm square aluminum channel filled with acrylic resin (OrthoJet BCA, Lang Dental, Wheeling IL, USA) with the femoral necks oriented at 15 degrees of internal rotation relative to the embedding block faces. Alignment consistency across samples was accomplished using a custom-made alignment fixture. The samples used in this study were a subset of those examined previously (17), limiting the analytic sample to those with body weight data.

### NanoComputed tomography images

Whole proximal femora were scanned using nanoCT at 27 µm voxel size, 110 kV, 200 µA, and a 0.762-mm aluminum filter, as described previously (17). A calibration phantom containing air, water, and a hydroxyapatite mimicker (1.69 mg/cc; Gammex, Middleton, WI, USA) was included in each scan to convert attenuation grayscale values to Hounsfield units. Image volumes were reconstructed using datos|x software (GE Inspection Technologies, LP, Skaneateles, NY, USA). Each volume was digitally rotated 15 degrees to account for anteversion using Dragonfly software (Comet Technologies, Montreal, QC, Canada). Two fully convolutional neural networks (FCNNs) were used; one to segment bone from background (*Dice = 0.983 ± 0.016*) and another to segment cortical from trabecular tissue (*Dice = 0.977 ± 0.023*), as validated previously (17). Both FCNNs were trained and validated on a sample of 30 white female cadaveric donors ranging in age from 36 to 89+ years, spanning the full spectrum of age-related cortical structural changes including superior cortical thinning to the width of a single trabecula. Each segmented image was manually reviewed for thresholding quality and cortical-trabecular segmentation accuracy, with the boundary defined by established protocols, and manual corrections were made for any false-positive or false-negative voxels. The delineation between cortical and trabecular bone was based on the approach described by Bell et al. (18, 19); the cortical:trabecular FCNN was trained to segment dense, low-porosity compact regions of cortical bone and higher-porosity endocortical regions that, while visually similar to trabecular bone, are anatomically distinct and reflect the progressive increase in resorption within the Haversian canals adjacent to the endocortical surface (20, 21) (**Figure 1**). The cortical:trabecular FCNN was not trained to segment based on a specific porosity value but rather on the pattern and continuity of similar structural features characteristic of tissue circumscribing the cross-section. The FCNN was trained to segment cortical tissue in the superior region that is as thin as a single trabecula and to simultaneously segment the thick cortical tissue in the inferior region.

Three femoral neck volumes of interest (FN VOIs) were generated (**Figure 1**): a 15 mm wide VOI located at the base of the FN, a 15 mm wide VOI centered at the narrow neck region, and a 3 mm wide VOI centered at the narrow neck region. The narrow neck site was identified as the location within the femoral neck having the smallest width perpendicular to the hip axis length. The two 15 mm wide VOIs were anatomically located to be similar to those of FN regions of interest defined by two widely used commercial manufacturers. The 3 mm wide VOI was consistent with the size and anatomical location of the region used in the HSA algorithm (16). Reliability of the FN VOI extraction process was confirmed with an intraclass correlation coefficient (ICC) of 0.9997 for area and 0.988 for bone mineral content (*BMC*) (17). Average cross-sectional cortical area (*Ct.Ar*), total subperiosteal area (*Tt.Ar*), trabecular *BV/TV*, superior-inferior outer diameter, and superior-inferior internal diameter were determined for the 3 mm VOI (**Figure 1**). The fraction of total bone mass (*BMC*) attributed to cortical and trabecular tissues was calculated for the three VOIs for each femur. Cross-sectional circularity was calculated for the narrow neck site as the ratio of the superior-inferior to the anterior-posterior periosteal diameter.

### PseudoDXA images

PseudoDXA images were generated for each of the three FN VOIs described above using a custom written Dragonfly plug-in. Briefly, a two-dimensional bone density map (pseudoDXA image) was generated by projecting all bone voxels onto a planar region of interest (ROI) with the proximal femur anteverted 15 degrees, consistent with standard positioning during a routine DXA image acquisition. PseudoDXA images were created for cortical bone, trabecular bone, and the combination of both. PseudoDXA parameters, including the projected ROI area (pseudoDXA area), total number of bone voxels (pseudoDXA BMC), and the total number of bone voxels per area (pseudoDXA aBMD), were calculated. Average and minimum FN widths (*FNW*) were measured directly from the pseudoDXA images. PseudoDXA parameters were significantly correlated with corresponding parameters measured from a commercial Hologic DXA system in a validation sample of 19 White female donors (22): BMC (*R^2^ = 0.95*, *p<0.0001*), areal BMD (*R^2^ = 0.92*, *p<0.0001*), and ROI area (*R^2^ = 0.58*, *p<0.0001*).

### HSA algorithms

The hip structure analysis (HSA) estimates of FN morphology were calculated at the narrow neck location using the algorithms and methods described previously (16). The HSA structural measures included the following:


$$R_{outer}=FNW/2.$$

$$A=\sum \left(t_{i}\;p_{s}\right)$$

Rinner=(Router2− fc(A / π))12

$$BR=R_{outer}/\left(R_{outer}-\;R_{inner}\right)$$

$$Ct.Ar=\pi \;\left({R_{outer}}^{2}-\;{R_{inner}}^{2}\right)$$

$$Ct.Th=R_{outer}-\;R_{inner}$$

where *FNW* is the femoral neck width, *R_outer_* is the outer radius of the femoral neck cross-section, *t_j_* is the linear attenuation value at pixel *j* along the mass profile, *p_s_* is the pixel size, *A* is the cross-sectional area of bone minus voids, spaces, and marrow cavity (equivalent to *CSA*), *BR* is the buckling ratio, *Ct.Ar* is cortical area, *R_inner_* is the endocortical radius, and *Ct.Th* is cortical thickness. It is important to distinguish between cross-sectional area (*CSA*) in the HSA program and cortical area (*Ct.Ar*). For purposes of this investigation and to be consistent with the HSA algorithms, *CSA* is derived from the X-ray attenuation profile without simplifying assumptions and represents the total area occupied by bone tissue at a given cross-section, excluding voids, marrow spaces, and other cavities that do not contribute meaningfully to load-bearing capacity and *CSA* and *A* are used interchangeably (16). *Ct.Ar* is the cross-sectional area occupied by cortical bone and is calculated assuming a circular profile with a uniformly thick cortex.

The HSA structural measures were calculated in two ways: first, using a fixed proportion of BMC attributable to cortical bone (fixed *f_c_* = 0.6) and second, using the proportion of BMC attributable to cortical bone measured from the 3 mm nanoCT volume centered at the narrow neck site (measured *f_c_*). For comparison to actual structural values, *Ct.Ar*, *R_inner_*, and the buckling ratio were measured directly from the 3 mm nanoCT volume located at the narrow neck site (**Figure 1**).

### Analysis

Data were expressed as mean ± standard deviation (SD). Normality was confirmed using the D’Agostino and Pearson test. Data were reported for females and males separately. Independent t-tests were used to compare traits between female and male donors. Data were adjusted to the average age of the female and male donors (61.6 years) when FN traits were directly compared between sexes. For all analyses, the 3 mm VOI was used for the HSA algorithms to be consistent with the protocols described previously (16). Likewise, comparisons to the actual structural measures were conducted using traits measured from the 3 mm VOI located at the narrow neck site. Values measured directly from the nanoCT images were the standard against which HSA parameters calculated using fixed *f_c_* and measured *f_c_* were compared. All statistical analyses were conducted using Prism (version 10, GraphPad Software, LLC, Boston, MA, USA), except as noted.

The first analysis (*f_c_ Variation)* determined the extent to which *f_c_* varied among individuals, with body size, and with age. All analyses were conducted separately for females and males. To test how the measured *f_c_* values varied along the femoral neck, a linear regression analysis was conducted between the *f_c_* measured for the 3 mm VOI and the *f_c_* measured for the 15 mm VOIs corresponding to two commercially available DXA systems (**Figure 1**). A repeated measures (RM) one-way ANOVA with Geisser-Greenhouse correction was conducted separately for females and males to assess whether *f_c_* differed across the three VOI locations. A correlation matrix was established among *f_c_*, age, weight, pseudoDXA BMC, and FNW to assess the associations among these parameters; *f_c_* and pseudoDXA BMC were assessed for the 3 mm VOI. We examined associations with age, weight, BMC, and FNW because these parameters are available clinically. Pairwise correlations between *f_c_* and demographic and imaging variables (**Table 1**) were computed as an exploratory screening step to inform variable selection for multivariable regression modeling. To account for multiple comparisons within this screening step, p-values were adjusted using the Benjamini-Hochberg false discovery rate (FDR) procedure applied separately within each sex (15 pairwise tests per sex) using p.adjust() in R (v 4.6.0, The R Foundation for Statistical Computing, Vienna, Austria). Associations with FDR-adjusted *q<0.05* were considered statistically significant for the purpose of variable selection. Finally, a multivariable linear regression analysis was conducted between *f_c_* and age, weight, BMC, and FNW to identify significant predictors of *f_c_*. Variance inflation factors (*VIFs*) were calculated to assess the extent to which multicollinearity inflated the variance of the estimated regression coefficients.

**Table 1:** Demographic characteristics and femoral neck morphology for female and male donors.

Parameter	Female (n=32)	Male (n=30)	p-value^a^
Age (years)	65.5 ± 20.6	57.6 ± 21.4	0.144
Weight (kg)	67.3 ± 26.2	85.3 ± 23.8	0.007
Height (m)	1.61 ± 0.09	1.76 ± 0.07	<0.001
BMI (kg/m^2^)	25.9 ± 8.8	27.5 ± 7.6	0.474
FNW (mm)	29.5 ± 1.8	34.9 ± 2.3	<0.001
FNW, age-adjusted (mm)	29.5 ± 1.8	34.9 ± 2.2	<0.001
f_c_	0.48 ± 0.09	0.42 ± 0.08	0.010
f_c_, age-adjusted	0.47 ± 0.08	0.42 ± 0.08	0.024
Circularity (SI/AP)	1.00 ± 0.09	1.00 ± 0.07	0.969
Circularity (SI/AP), age-adjusted	1.00 ± 0.09	1.00 ± 0.06	0.826

Values are mean ± SD. BMI, body mass index; FNW, femoral neck width; f_c_, cortical fraction; SI, superior-inferior; AP, anterior-posterior. ^a^Independent t-test for unadjusted comparisons; independent t-test on age-adjusted values.

The second analysis (*Effects of fixed versus measured f_c_ on morphology estimates*) determined the extent to which assuming a fixed *f_c_* affected estimates of morphology using the HSA algorithms compared to using the measured *f_c_*. First, a Bland-Altman analysis was conducted to assess the agreement between the HSA estimates using the fixed *f_c_* versus the measured *f_c_* values. The bias and 95% limits of agreement were calculated for this analysis. Second, a linear regression analysis was conducted comparing the structural values estimated from HSA using fixed *f_c_* and measured *f_c_* with those measured directly from the corresponding nanoCT images. The slopes and elevations of these two regressions were compared using the ‘Compare lines’ function in Prism, which tests for differences using an F-test and is mathematically equivalent to ANCOVA. For each regression, we also calculated Lin’s concordance correlation coefficients, which assess the agreement (concordance) between two measures of a variable (i.e., HSA estimate versus nanoCT value) (R v 4.6.0). If the HSA estimates for *R_inner_*, *Ct.Ar*, and *BR* were identical to those measured from the nanoCT image, then the regression would approximate the identity line (i.e., *x = y*) and Lin’s concordance correlation coefficient would be 1. Differences in concordance between the regression of the HSA estimates using fixed *f_c_* versus nanoCT-derived values and the regression of the HSA estimates using measured *f_c_* versus nanoCT-derived values were considered significant when the 95% confidence intervals did not overlap.

The third analysis (*Age-related errors in morphology estimates)* assessed how the errors associated with using fixed *f_c_* and measured *f_c_* to estimate *R_inner_*, *Ct.Ar*, and *BR* differed as a function of age. Female and male samples were sorted into younger (≤65 years of age) and older (>65 years of age) age groups. Sorting the samples using 65 years of age split the samples into relatively similar-sized subgroups and coincided with the age when bone health monitoring tends to begin. The structural estimates were compared to the traits measured from nanoCT to assess the errors when estimating the parameters using fixed *f_c_* and measured *f_c_*. The errors were compared between younger and older subgroups using a t-test to determine if the errors differed between age groups. The errors were also compared using paired t-tests for each of the age subgroups to determine if using the measured *f_c_* reduced the errors compared to using a fixed *f_c_*. Analyses were conducted for females and males separately.

The fourth analysis (*VOI mismatch error estimation*) estimated the additional error introduced when applying a 3 mm narrow neck VOI-derived *f_c_* correction to commercial HSA systems that average over a 15 mm region. HSA structural parameters (*R_inner_*, *Ct.Th*, *Ct.Ar*, and *BR*) were computed for each specimen under three *f_c_* scenarios: *f_c_* measured at the 3 mm narrow neck VOI, *f_c_* measured at the 15 mm narrow neck VOI, and *f_c_* measured at the 15 mm base of FN VOI, using the equations described in the HSA Algorithms section. Percent errors for each 15 mm scenario were computed relative to estimates derived using *f_c_* from the 3 mm narrow neck VOI, which served as the reference standard consistent with Analyses 2 and 3. Mismatch errors are additionally reported as a proportion of the fixed *f_c_* error magnitude to contextualize their clinical relevance. Differences in *f_c_* between the 3 mm narrow neck VOI and each 15 mm VOI were tested with one-sample t-tests against a null hypothesis of zero difference, conducted separately for females and males.

## Results

### Analysis 1: *f_c_* variation

The demographic data, *FNW*, measured *f_c_* values, and circularity are shown in **Table 1**. Female and male donors spanned 18 to over 89 years of age with group averages of 65.5 ± 20.6 years and 57.6 ± 21.4 years for females and males (*p=0.144*, t-test), respectively. Males were heavier (*p=0.007*, t-test) and taller (*p* < 0.0001) but showed no difference in *BMI* (*p=0.474*, t-test) compared to females. Males had wider *FNW* (*p<0.001*, t-test) compared to females, as expected. Mean values for *f_c_* were 0.48 ± 0.09 for females and 0.42 ± 0.08 for males, which differed significantly between sexes (*p=0.010*, t-test) even after adjusting for age (*p=0.024*, t-test). The circularity, which was calculated as the ratio of periosteal border diameters in the superior-inferior and anterior-posterior directions, was 1.00 ± 0.09 for females and 1.00 ± 0.06 for males, which did not differ between sexes after adjusting for age (*p=0.826*, t-test).

The *f_c_* values calculated for the 3 mm NN region correlated significantly with the *f_c_* values calculated for two 15 mm wide VOIs (**Figure 2**), though *f_c_* differed significantly across the three VOI locations for both females and males (Females: *F(1.12, 34.73)=59.63*, *p<0.0001*; Males: *F(1.19, 34.39)=46.48*, *p<0.0001*; RM one-way ANOVA with Geisser-Greenhouse correction). The linear regressions showed significant differences in the elevations between the base and narrow neck VOIs, with the *f_c_* values calculated for the FN base location being larger than those calculated for the narrow neck and 3 mm VOIs for both females and males (*p<0.0001*, paired t-test).

**Figure 2:**
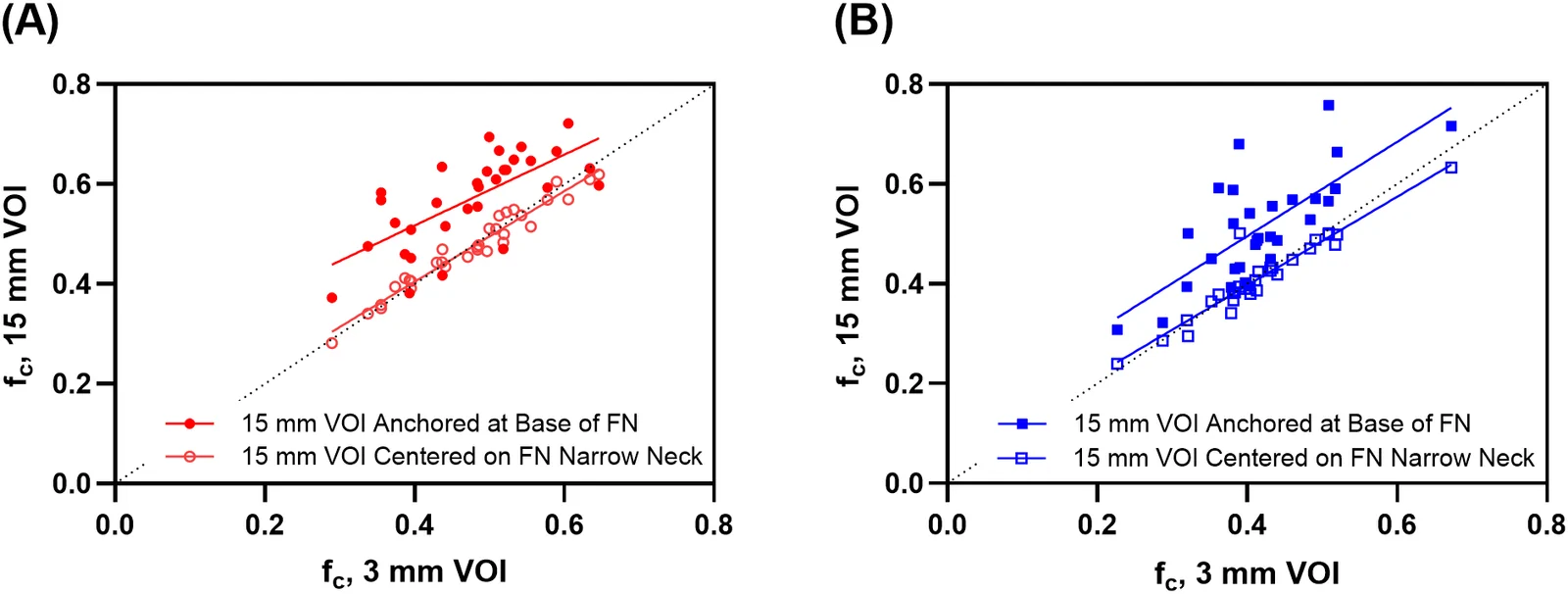
Associations among measured cortical fraction (*f_c_*) values across femoral neck VOIs for females **(A)** and males **(B)**. The *f_c_* measured from the 3 mm narrow neck VOI is plotted against *f_c_* from the 15 mm base of FN VOI (filled symbols) and the 15 mm narrow neck VOI (open symbols). The dashed line is the line of identity. *f_c_* at the FN base was significantly greater than at the narrow neck and 3 mm VOIs for both sexes (*p<0.0001*, ANCOVA). *f_c_* differed significantly across the three VOI locations for both females and males (*p<0.0001*, RM one-way ANOVA with Geisser-Greenhouse correction).

The correlation matrix showed associations between *f_c_* and clinically available parameters including age, weight, *BMC*, and *FNW* (**Table 2**). For females, significant associations were found between *f_c_* and age (*p=0.004*). For males, significant associations were found between *f_c_* and weight (*p<0.001*). After Benjamini-Hochberg FDR correction (15 pairwise tests per sex, *α = 0.05*), the two primary associations remained significant: *f_c_* vs. Age in females (raw *p=0.004*, *q = 0.010*) and *f_c_* vs. Weight in males (raw *p=0.0004*, *q=0.006*). In females, no additional *f_c_* correlations were lost, as all correlations significant at the uncorrected threshold survived FDR correction. In males, *f_c_* vs. pseudoDXA BMC was nominally significant at the uncorrected threshold (*p=0.044*) but did not survive FDR correction (*q=0.165*); this variable did not enter the final male multivariable model, and no downstream conclusions are affected by this correction. A multivariable linear regression analysis was performed separately for females and males to identify significant independent predictors of *f_c_* (**Table 3**). For females, age and *BMC* were significant predictors with adjusted *R^2^ = 0.361* (p=0.003) for the overall regression. For males, weight remained the only significant predictor in the multivariable regression model with adjusted *R^2^ = 0.402* (*p=0.002*) for the overall regression. Variance inflation factors (*VIFs*) were less than 5 confirming little to moderate correlation among predictor variables.

**Table 2:** Associations among measured f_c_ and clinically available parameters.

A. Females						
Parameter	*f_c_*	Age	Height	Weight	*R_outer_*	pseudoDXA *BMC*
*f_c_*	1.00	**0.49**	-0.12	0.03	0.10	0.02
Age		1.00	**-0.51**	-0.27	0.07	**-0.57**
Height			1.00	**0.49**	**0.50**	**0.66**
Weight				1.00	0.32	**0.68**
*R_outer_*					1.00	**0.47**
pseudoDXA *BMC*						1.00

B. Males						
Parameter	*f_c_*	Age	Height	Weight	*R_outer_*	pseudoDXA *BMC*
*f_c_*	1.00	0.20	0.03	**-0.62**	-0.17	-0.37
Age		1.00	0.00	-0.07	0.08	**-0.47**
Height			1.00	0.14	**0.49**	0.19
Weight				1.00	0.08	0.28
*R_outer_*					1.00	0.19
pseudoDXA *BMC*						1.00

Bold font indicates significant associations (p<0.05). *f_c_*, cortical fraction; *R_outer_*, outer radius = *FNW*/2; *FNW*, femoral neck width; pseudoDXA *BMC*, bone mineral content derived from simulated DXA images generated by projection of nanoCT data. Pairwise correlations were computed as an exploratory screen (15 tests per sex) with Benjamini-Hochberg FDR correction applied. Bold font indicates associations surviving FDR correction (q< 0.05). In males, the *f_c_* vs. pseudoDXA BMC correlation was significant at the uncorrected threshold (p = 0.044) but did not survive FDR correction (q = 0.165) and is not bolded; this variable did not enter the final male multivariable model.

**Table 3:** Multivariable linear regression analysis between measured *f_c_* and clinically available parameters.

A. Females						
Parameter	Estimate	Standard Error	95% CI	t	p-value	VIF
Intercept	0.380	0.217	-0.065 to 0.825	1.752	0.091	
Age (years)	**0.004**	**0.001**	**0.002 to 0.006**	**4.577**	**<0.0001**	2.012
Weight (kg)	-0.0007	0.001	-0.002 to 0.001	0.9767	0.337	1.990
FNW (mm)	-0.014	0.009	-0.033 to 0.005	1.530	0.138	1.651
BMC (g)	**0.234**	**0.080**	**0.068 to 0.399**	**2.901**	**0.007**	3.923
			**Overall regression**	**R_adj_^2^ = 0.361**	**p=0.003**	

B. Males						
Parameter	Estimate	Standard Error	95% CI	t	p-value	VIF
Intercept	0.630	0.162	0.295 to 0.964	3.885	0.001	
Age (years)	0.0002	0.001	-0.001 to 0.001	0.463	0.648	1.339
Weight (kg)	**-0.002**	**0.0004**	**-0.002 to -0.001**	**3.460**	**0.002**	1.088
FNW (mm)	0.0001	0.005	-0.010 to 0.010	0.014	0.989	1.086
BMC (g)	-0.058	0.035	-0.130 to 0.013	1.680	0.106	1.484
			**Overall regression**	**R_adj_^2^ = 0.402**	**p=0.002**	

Bold font indicates significant predictors (*p<0.05*). *BMC*: bone mineral content; *FNW*: femoral neck width; *VIF*: variance inflation factor; *t*: t-statistic (*Estimate/Standard Error*).

To better understand the sex-specific associations among *f_c_*, age, and weight observed in the multivariable models, follow-up linear regression analyses were conducted using the amount of cortical and trabecular bone versus age and weight. Cortical and trabecular tissues can be segmented in pseudoDXA images, and the amount of each tissue type is measured as the number of voxels. The total number of bone voxels was not included in the ANCOVA regression comparisons but is shown for reference purposes and to provide context for how *f_c_* varies with age and weight (**Figures 3A, B, D, E**). For the age associations (**Figures 3A–C**), both females and males showed significant negative associations between the number of trabecular voxels and age (*R^2^ = 0.525*, *p<0.0001* females; *R^2^ = 0.175*, *p=0.022* males) and total voxels and age (*R²=0.329*, *p=0.0006* females; *R²=0.218*, *p=0.009* males), but the associations between the number of cortical voxels and age were not significant (*R^2^ = 0.028*, *p=0.356* females; *R^2^ = 0.036*, *p=0.312* males). The resulting *f_c_* values showed a positive association with age for females (*R^2^ = 0.243*, *p=0.004*) but not males (*R^2^ = 0.042*, *p=0.280*). To understand the association between *f_c_* and weight (**Figures 3D–F**), we first adjusted the number of cortical and trabecular voxels for age by computing residuals from a linear regression of voxel counts on age, separately for females and males. For females, the number of age-adjusted cortical (*R²=0.273*, *p=0.002*), trabecular (*R²=0.314*, *p=0.0009*), and total voxels (*R²=0.452*, *p<0.0001*) all showed significant positive associations with weight, with a significant difference in elevations (*p=0.020*, ANCOVA) but no difference in slopes (*p=0.702*, ANCOVA). In contrast, the number of age-adjusted cortical and trabecular voxels showed divergent associations with weight for males (difference in slopes: *p=0.0004*, ANCOVA), with the number of cortical voxels showing a significant negative association with weight (*R^2^ = 0.144*, *p=0.042*) and the number of trabecular voxels showing a positive association with weight (*R^2^ = 0.263*, *p=0.005*), while total voxels did not show a significant association with weight (*R²=0.077*, *p=0.146*). Given the sex-specific regressions between cortical and trabecular voxels versus weight, it was not surprising that males (*R^2^ = 0.384*, *p=0.0003*) but not females (*R^2^ = 0.036*, *p=0.298*) showed a significant association between *f_c_* and weight (slopes, p=0.002, ANCOVA).

**Figure 3:**
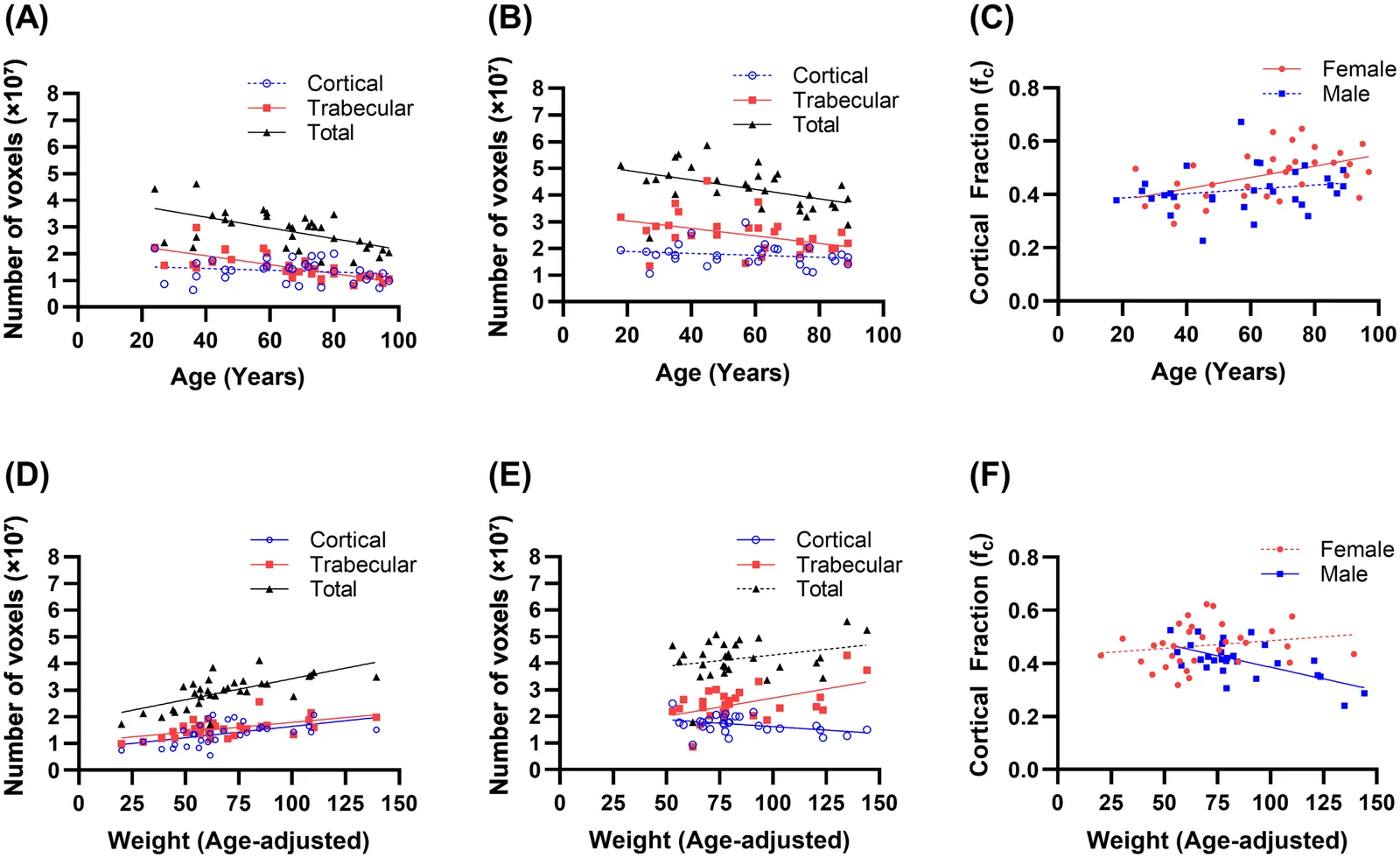
Associations between the number of cortical, trabecular, and total bone voxels and age for females **(A)** and males **(B)**, cortical fraction (*f_c_*) versus age for females and males **(C)**, age-adjusted cortical, trabecular, and total bone voxels versus age-adjusted weight for females **(D)** and males **(E)**, and fc versus age-adjusted weight for females and males **(F)**. Linear regression lines are shown for all data series; dashed lines indicate non-significant associations.

### Analysis 2: effects of fixed versus measured *f_c_* on morphology estimates

In the second analysis, we tested whether using the measured *f_c_* in the HSA algorithms improved estimates of FN structural parameters compared to using a fixed *f_c_*. The Bland-Altman analysis showed that substituting fixed *f_c_* for measured *f_c_* systematically underestimated *R_inner_* and *BR* and overestimated *Ct.Ar* in both females and males, with larger bias in males and wider limits of agreement or R_inner_ and *Ct.Ar* in males (**Table 4**). Linear regression analysis showed significant correlations between measured *R_inner_* and both HSA estimates for both females and males (**Figure 4**). A comparison of the linear regressions showed significantly lower elevations for the fixed *f_c_* regression for females (*p=0.007*, ANCOVA) and males (*p<0.0001*, ANCOVA). Both *BR* estimates correlated significantly with measured *BR* for females and males. The slopes differed significantly between estimates (females: *p=0.002*; males: *p=0.007*, ANCOVA), with fixed *f_c_* estimates generally falling below those using measured *f_c_*. Both Ct.Ar estimates correlated significantly with measured *Ct.Ar* for females and males. The elevations differed significantly between estimates (females: *p<0.0001*; males: *p<0.0001*, ANCOVA), with fixed *f_c_* estimates generally falling well above those using the measured *f_c_*. Lin’s concordance correlation coefficients were generally improved when the measured *f_c_* values were used to estimate *R_inner_*, *Ct.Ar*, and *BR* (**Table 5**). Although using measured *f_c_* generally produced estimates more closely aligned with nanoCT-measured values, a significantly greater improvement in concordance based on non-overlapping 95% confidence intervals for Lin’s concordance correlation coefficients was observed for *R_inner_* and *Ct.Ar* in males.

**Table 4:** Bland-Altman bias and 95% limits of agreement comparing HSA estimates using fixed *f_c_* versus measured *f_c_*.

Trait	Females Bias (95% LOA)	Males Bias (95% LOA)
*R_inner_* (mm)	-0.26 (-0.67 to 0.15)	-0.51 (-1.11 to 0.08)
*Buckling Ratio*	-4.05 (-10.99 to 2.89)	-5.76 (-12.27 to 0.75)
*Ct.Ar* (mm^2^)	+22.71 (-12.59 to 58.01)	+51.70 (-6.16 to 109.6)

Bias = fixed *f_c_* estimate minus measured *f_c_* estimate. Positive values indicate overestimation by fixed *f_c_*; negative values indicate underestimation. LOA, limits of agreement.

**Figure 4:**
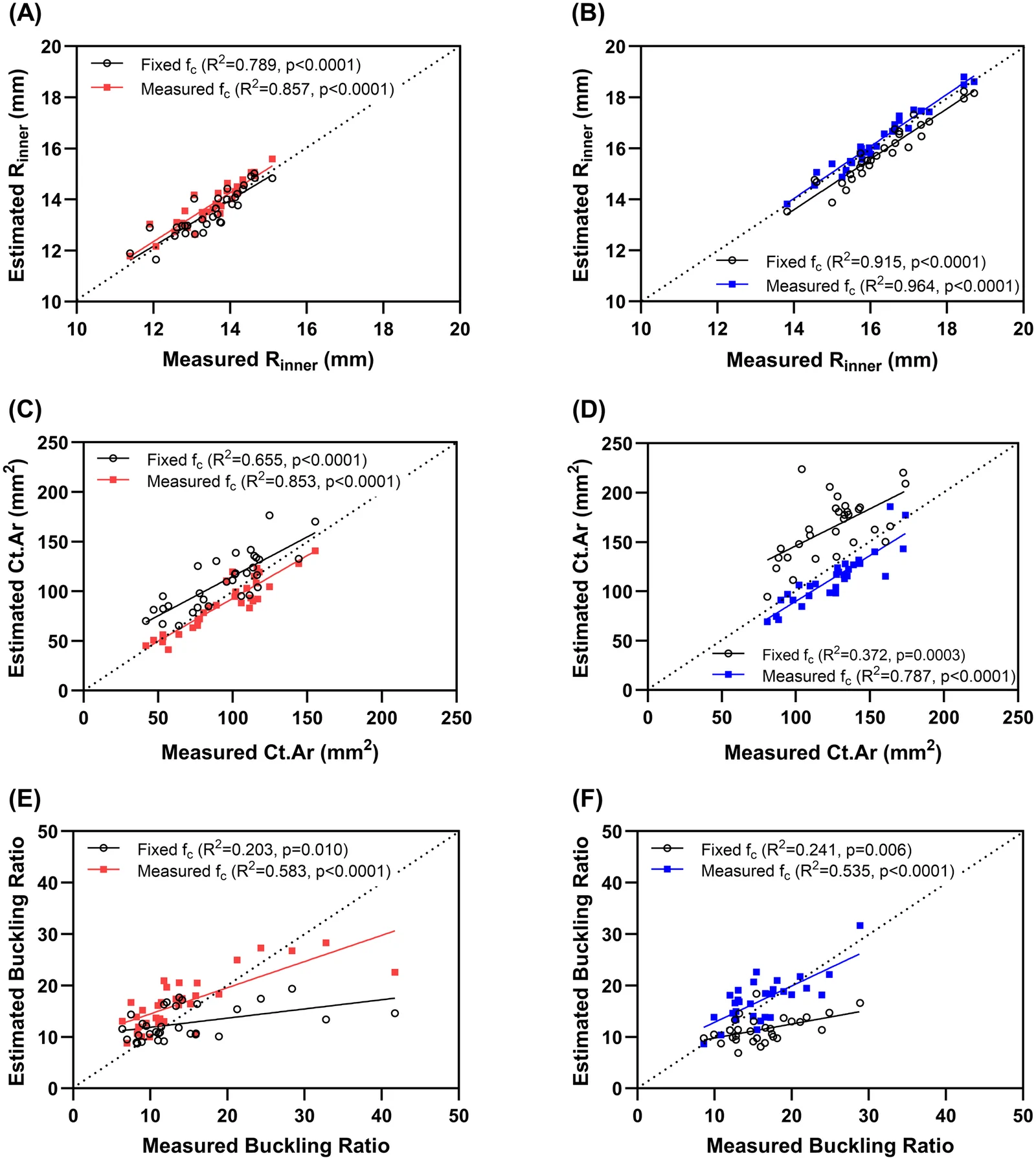
Linear regression of measured versus HSA-estimated *R_inner_* **(A, B)**, *Ct.Ar* **(C, D)**, and buckling ratio **(E, F)** for females **(A, C, E)** and males **(B, D, F)** using fixed *f_c_* (open circles, black line) and measured *f_c_* (filled squares, colored line). *R²* and *p*-values for each regression are shown in the legend. The dotted line is the identity line. ANCOVA comparisons: *R_inner_* – lower elevations for fixed *f_c_* in females (*p=0.007*) and males (*p<0.0001*); buckling ratio – steeper slopes for measured *f_c_* in females (*p=0.002*) and males (*p=0.007*); *Ct.Ar* – higher elevations for fixed *f_c_* in females (*p<0.0001*) and males (*p<0.0001*). Lin’s concordance correlation coefficients are provided in **Table 5**.

**Table 5:** Lin’s concordance correlation coefficients (CCC) for HSA estimates using fixed *f_c_* and measured *f_c_* compared to nanoCT-measured values.

A. Females		
Trait	Fixed *f_c_* CCC (95% CI)	Measured *f_c_* CCC (95% CI)
*R_inner_* (mm)	0.888 (0.783 to 0.943)	0.878 (0.777 to 0.935)
*Buckling Ratio*	0.296 (0.082 to 0.484)	0.663 (0.467 to 0.797)
*Ct.Ar* (mm^2^)	0.689 (0.495 to 0.818)	0.897 (0.806 to 0.947)

B. Males		
Trait	Fixed *f_c_* CCC (95% CI)	Measured *f_c_* CCC (95% CI)
*R_inner_* (mm)	0.895 (0.809 to 0.943)	0.979 (0.957 to 0.990)
*Buckling Ratio*	0.227 (0.054 to 0.386)	0.712 (0.486 to 0.849)
*Ct.Ar* (mm^2^)	0.297 (0.117 to 0.458)	0.800 (0.648 to 0.890)

CCC, concordance correlation coefficient; CI, confidence interval. Differences in concordance were considered significant when 95% CIs did not overlap. A significantly greater improvement for concordance with measured *f_c_* was observed for R_inner_ and Ct.Ar in males.

### Analysis 3: age-related errors in morphology estimates

The third analysis tested if using a fixed *f_c_* to estimate structural parameters showed greater errors in older (>65) individuals compared to younger (≤65) individuals and whether these errors were reduced when using measured *f_c_* values (**Table 6**). For *R_inner_*, the percent error differed significantly between older and younger females but not males. Using the measured *f_c_* significantly reduced the errors for males but not females. The errors for *BR* were substantial (16-37%) for females whether using fixed or measured *f_c_* values. In contrast, the large errors (23-28%) associated with estimating *BR* using fixed *f_c_* were significantly reduced to 9-10% when using measured *f_c_* values (*p<0.0001*, paired t-test) for males. Finally, using fixed *f_c_* values to estimate *Ct.Ar* resulted in large errors for both females (13-36%) and males (31-36%), and in both sexes these errors were significantly reduced to 5-10% when using measured *f_c_* values (*p≤0.001*, paired t-test). Errors associated with estimating structural parameters using fixed *f_c_* values were significantly different between younger and older women. *R_inner_* and *BR* were underestimated for younger women and overestimated for younger women. *Ct.Ar* was overestimated for both age groups, but the error was significantly larger for younger women. No significant difference in errors between younger and older women were found when using the measured *f_c_* to estimate structure. The errors in estimating structural parameters using fixed *f_c_* and the measured *f_c_* did not differ between younger and older men (t-test); however, *post-hoc* power analyses revealed low achieved power for these comparisons (5–27%), indicating that these null results should be interpreted cautiously and are most likely attributable to insufficient sample size rather than a true absence of age-related differences.

**Table 6:** Comparison of errors for females and males sorted by age into younger (≤65 years) and older (>65 years) subgroups.

A. Females				
Trait	Age Group (*n*)	% Error with fixed *f_c_*	% Error with measured *f_c_*	Fixed vs measured *f_c_* (paired t-test)
*R_inner_*	Younger (13)	**-1.9 ± 2.2**	1.4 ± 2.0	***p<0.0001***
	Older (19)	**1.6 ± 3.0**	2.6 ± 2.9	***p<0.0002***
	Age-effect (t-test)	***p=0.001***	*p=0.205*	
*BR*	Younger (13)	**-20.8 ± 24.9**	15.7 ± 28.9	***p<0.0001***
	Older (19)	**17.4 ± 34.7**	-37.1 ± 37.5	***p<0.0001***
	Age-effect (t-test)	***p=0.002***	p=0.094	
*Ct.Ar*	Younger (13)	**36.3 ± 21.9**	-8.0 ± 9.9	***p<0.0001***
	Older (19)	**12.6 ± 23.1**	-4.7 ± 12.0	***p=0.001***
	Age-effect (t-test)	***p=0.007***	*p=0.430*	

B. Males				
Trait	Age Group (*n*)	% Error with fixed *f_c_*	% Error with measured *f_c_*	Fixed vs measured *f_c_* (paired t-test)
*R_inner_*	Younger (18)	-3.1 ± 2.4	0.4 ± 1.4	***p<0.0001***
	Older (12)	-2.0 ± 1.8	0.6 ± 1.5	***p<0.0001***
	Age-effect (t-test)	*p=0.177*	*p=0.771*	
*BR*	Younger (18)	-27.9 ± 19.3	8.6 ± 21.8	***p<0.0001***
	Older (12)	-22.6 ± 22.2	9.5 ± 23.5	***p<0.0001***
	Age-effect (t-test)	*p=0.495*	*p=0.921*	
*Ct.Ar*	Younger (18)	35.9 ± 26.3	10.5 ± 8.7	**p<0.0001**
	Older (12)	31.0 ± 19.5	-8.1 ± 9.4	**p<0.0001**
	Age-effect (t-test)	p=0.592	p=0.495	

Data are shown as the mean % error ± SD, where % error = 100 × (estimated − measured)/measured. Bold font indicates significant differences (p<0.05). Age-effect: comparison between younger and older subgroups using an independent t-test.

### Analysis 4: VOI mismatch error estimation

The mean *f_c_* at the 3 mm narrow neck VOI did not differ significantly from *f_c_* at the 15 mm narrow neck VOI in either females *(+0.003 ± 0.020*, *t (*31)*=0.780*, *p=0.441*, one-sample t-test) or males (*+0.005 ± 0.027*, *t(29)=1.036*, *p=0.309*, one-sample t-test). Consequently, the additional error introduced by applying a 3 mm-derived *f_c_* correction to a commercial narrow neck system was negligible, representing 8–15% of the fixed *f_c_* error magnitude across structural parameters and both sexes. In contrast, *f_c_* at the 3 mm narrow neck VOI was significantly lower than *f_c_* at the 15 mm base of FN VOI in both females (*−0.095 ± 0.070*, *t(31)=7.674*, *p<0.0001*, one-sample t-test) and males (*−0.095 ± 0.074*, *t(29)=6.979*, *p<0.0001*, one-sample t-test). Applying a 3 mm-derived *f_c_* correction to a base of FN commercial system introduced mismatch errors representing 47–83% of the fixed *f_c_* error magnitude, indicating that only a portion of the available improvement would be recovered in that context.

## Discussion

The analyses described in the current study expand on our previous study by focusing on how the proportion of cortical and trabecular tissue mass varies among individuals and how this varying proportion affects algorithms used to estimate structural properties (7). The goal of this study was to critically evaluate HSA estimates of femoral neck structural measures for systematic errors. DXA images are a 2D projection of a 3D structure, and simplifying assumptions are needed to extract meaningful structural information. The HSA algorithms assume the femoral neck has a circular cross-section with a uniformly thick cortex that accounts for 60% of the total bone mineral content to estimate *R_inner_*, *BR*, and *Ct.Ar*. Using high-resolution nanoCT data as a means of comparison, we 1) investigated whether the ratio of cortical to total bone mineral content (*f_c_*) varied with sex, age, and body size; 2) determined errors in estimating femoral neck morphology resulting from the assumption of a fixed *f_c_*; and 3) determined errors in estimating femoral neck morphology with measured *f_c_* determined from nanoCT data. The primary source of error was the assumption that cortical bone accounts for 60% of the total mineral content. Analysis of proximal femur nanoCT images confirmed that *f_c_* measured at the narrow neck site is not fixed, but varies significantly with factors such as sex, age, weight, and anatomical site along the femoral neck. When compared to structural measures assessed from nanoCT volumes, the errors associated with using a fixed *f_c_* reached up to 36% for *Ct.Ar* in both sexes. Replacing fixed *f_c_* with measured *f_c_* significantly reduced errors for *Ct.Ar* and *R_inner_* in males and *Ct.Ar* in females. Thus, we showed that the fixed *f_c_* assumption is associated with significant errors in structural estimates that are age- and sex-specific.

One of the clearest findings from our data is that *f_c_* varies considerably between individuals, and that the mean narrow neck values of 0.48 for females and 0.42 for males, with individual values ranging from 0.23 to 0.67, are well below the 0.6 value that HSA assumes. The femoral neck is not a uniform cortical structure; rather, its composition varies depending on location along the neck axis. The cross-section is predominantly cortical near the femoral shaft, but the proportion of trabecular bone increases progressively toward the femoral head (23). A single fixed *f_c_* value will therefore fail to capture both the spatial heterogeneity of femoral neck composition and the considerable interindividual variation in cortical fraction that exists within a population. The fact that our mean values fall below 0.6 in both sexes likely reflects the mixed cortical-trabecular character of the mid-neck region where the narrow neck measurement is taken, rather than a fundamental disagreement with prior estimates made at more shaft-adjacent locations along the femoral neck.

The origin of the *f_c_* = 0.6 value itself warrants examination. In Beck et al. (11), the assumption that 60% of measured mineral mass at the narrow neck is cortical is attributed to two *in vitro* studies: Kuiper et al. (24) and Bell et al. (19). However, neither study directly supports this value as applied to the narrow neck ROI used in HSA. Kuiper et al. used quantitative computed tomography (QCT) in 20 embalmed cadaveric femoral necks and reported that at the smallest cross-sectional slice, corresponding most closely to the HSA narrow neck ROI, trabecular bone constituted 33% of total bone mass, implying a cortical fraction of 0.67, not 0.6 (24). A cortical fraction closer to 0.6 in the Kuiper data corresponds to a location approximately 8 millimeters proximal to the narrow neck, toward the femoral head. Bell et al. used histomorphometry in cadaveric femoral neck biopsies to characterize regional cortical and cancellous bone distribution by octant, reporting geometric area fractions rather than mineral mass fractions (19). Because cortical bone is considerably denser than trabecular bone, the same geometric area of cortical bone contributes disproportionately more to total mineral mass than trabecular bone of equal area, meaning the mineral mass fraction of cortical bone will always exceed its geometric area fraction. Bell’s histomorphometric data cannot therefore be directly used to establish the mineral mass fraction that HSA requires without additional density assumptions that Bell et al. did not provide. Neither study was designed specifically to establish a mineral mass fraction for DXA-based structural analysis at the narrow neck, and neither used measurements at precisely the 3 mm narrow neck VOI that the HSA software analyzes. The empirical basis of the *f_c_* = 0.6 assumption, as applied to the 3 mm narrow neck ROI used in HSA, does not appear to be well established by the studies cited as its source. To our knowledge, the present study provides the first systematic evaluation of this assumption using high-resolution nanoCT data across a large cadaveric sample spanning a wide age range and finds mean narrow neck *f_c_* values of 0.48 for females and 0.42 for males, both substantially below 0.6.

Differences in segmentation approach, specifically our use of density-based FCNN segmentation at 27 µm resolution versus the QCT thresholding used by Kuiper et al. and the histomorphometric area fractions reported by Bell et al., may partially account for this discrepancy (19, 24). The circularity of the narrow neck cross-section was confirmed at 1.00 ± 0.09 for females and 1.00 ± 0.06 for males, confirming that the circular cross-section assumption held while the *f_c_* assumption did not. The Kuiper et al. data show that *f_c_* changes approximately 2.5% per mm along the femoral neck axis, and our analyses confirm that this spatial variation is detectable even within the narrow neck region itself, with *f_c_* differing significantly across the three VOI locations in both females and males (Females: *F(1.12, 34.73)=59.63*, *p<0.0001*; Males: *F(1.19, 34.39)=46.48*, *p<0.0001*; RM one-way ANOVA with Geisser-Greenhouse correction). Examination of cortical and trabecular voxels separately, afforded by pseudoDXA images and not available in clinical DXA, provided a deeper understanding of why assuming a fixed *f_c_* is problematic for the femoral neck. For the age associations (**Figures 3A–C**), both females and males showed significant negative associations between the number of trabecular voxels and age, while cortical voxels showed no significant age association in either sex. However, females appear to have proportionally less trabecular bone than males at all ages, so as trabecular bone declines with age, the cortical fraction rises, producing a significant positive association between *f_c_* and age in females (*R²=0.243*, *p=0.004*) that was not observed in males (*R²=0.042*, *p=0.280*). Population-based QCT data show that trabecular vBMD at the femoral neck declines by more than 50% over life in women compared with approximately 45% in men, with declines beginning before midlife, while cortical area at the femoral neck is relatively preserved in both sexes (25). Longitudinal QCT data further show that women lose cortical thickness and cortical vBMD at the femoral neck more rapidly than men, yet these losses are only weakly reflected in standard DXA-derived femoral neck aBMD (26), which helps explain why HSA algorithms relying on a fixed *f_c_* assumption cannot account for the age-related shifts in cortical fraction documented in the present study. This age association was not seen for males, where similar cortical and trabecular declines produce no net association between *f_c_* and age, consistent with more gradual, non-event-driven bone loss in men (27).

This study provided new insight into sex-specific differences in weight associations (**Figures 3D–F**). For females, larger body weights were accommodated with both greater trabecular and cortical bone, which explains why *f_c_* does not show a significant association with weight for females. However, for males, larger body weights were supported by an increase in trabecular bone but not cortical bone, which explains why *f_c_* decreased with weight for males. Heavier men had a proportionally lower percentage of bone mineral content occupied by cortical bone, suggesting different sex-specific skeletal loading strategies to accommodate higher body weight, a novel observation enabled by the pseudoDXA approach used here. Note that analyses were conducted within the observed weight range and do not include obese individuals.

Errors were large when using fixed *f_c_*. For females, errors in Ct.Ar ranged from 12.6% in older to 36.3% in younger females, and BR errors ranged from 17.4% in older to 20.8% in younger females, with younger females showing underestimation and older females showing overestimation of *BR*. For males, errors in *Ct.Ar* ranged from 31.0–35.9% and *BR* errors ranged from 22.6–27.9%. The Bland-Altman analysis showed that substituting fixed *f_c_* for measured *f_c_* systematically underestimated *R_inner_* and *B*R and overestimated *Ct.Ar* in both females and males, with larger bias in males and wider limits of agreement for R_inner_ and *Ct.Ar* in males (**Table 4**). Errors for *R_inner_* were smaller but compound into *Ct.Ar* estimates via squared terms in the algorithm, amplifying their effect on downstream structural parameters.

Using measured *f_c_* significantly reduced errors for *Ct.Ar* in both sexes and for *BR* in males, but *BR* errors remained large in females (15.7–37.1%) even with measured *f_c_*. Errors associated with using a fixed *f_c_* differed significantly between younger and older females but not males (**Table 6**), with younger females most affected, as Ct.Ar was overestimated by 36.3% because their relatively high trabecular content pulls measured *f_c_* well below 0.6. Using measured *f_c_* eliminated the age effect on errors in females and substantially reduced errors for all traits in males. Lin’s concordance correlation coefficients showed significantly greater improvement in concordance, based on non-overlapping 95% confidence intervals, for *Ct.Ar* and *R_inner_* in males (**Table 5**). The persistence of large *BR* errors in females despite using the correct *f_c_* value, compared with *BR* errors of 9-10% in males with measured *f_c_* (**Table 6**) suggests that *f_c_* is not the only source of systematic error in the *BR* algorithm for females, and that a second structural assumption requires examination.

The *BR* calculation assumes not only a fixed *f_c_* but also that the cortex is uniformly thick around the circumference of the femoral neck. This uniform cortical thickness assumption is less accurate in females than in males. Using CT in 77 proximal femurs spanning ages 20–95 years, Mayhew et al. showed that the femoral neck cortex is markedly asymmetric, with the inferior cortex substantially thicker than the superolateral cortex at all ages, and that this asymmetry increases with age more rapidly in females than males with superior and superoposterior cortical thickness declining 6.4% per decade in women compared with significantly smaller effects in men (28). Similarly, Bell et al. demonstrated using histomorphometry that the inferior cortex is disproportionately thick relative to the superior cortex, with a significant sex difference in cortical bone proportion specifically in the inferoanterior region, and Carpenter et al. confirmed using QCT in the AGES-Reykjavik cohort that females have a relatively thicker inferior cortex despite having smaller femoral necks overall (19, 27). When the inferior cortex is disproportionately thick, the actual *BR*, which reflects the true distribution of bone around the neutral axis, will be lower than the algorithm predicts, meaning the femoral neck is stronger in bending than HSA estimates even when *f_c_* is correctly specified. The HSA algorithm cannot account for this circumferential asymmetry in cortical thickness because it derives *BR* from a concentric circular model, and this limitation is more consequential in females than in males. These prior findings support the hypothesis that the uniform cortical thickness assumption is less valid in females than in males, though this was not directly tested in the present dataset.

In males, the more uniform circumferential distribution of cortical bone means that the uniform *Ct.Th* assumption holds more closely, and correcting *f_c_* alone is therefore sufficient to substantially reduce errors. The residual *BR* errors in females are not a failure of the *f_c_* correction but are consistent with the hypothesis that the uniform *Ct.Th* assumption introduces additional error in females; quantifying this contribution directly and developing methods to account for it using clinically available imaging represents an important direction for future work, particularly for female fracture risk assessment.

Beyond the residual female *BR* errors, the age-stratified analysis revealed additional clinically important patterns in the errors associated with fixed *f_c_*. Errors associated with using a fixed *f_c_* were significantly greater in younger females than in older females for both *Ct.Ar* and *BR* (**Table 6**), while no significant age effect was observed in males. This pattern is mechanistically consistent with the *f_c_*–age association described above: younger females have proportionally more trabecular bone, pulling their measured *f_c_* further below 0.6 and therefore producing larger errors when the fixed value is applied. The practical consequence is that younger women in whom the *f_c_* divergence from 0.6 is greatest are also the individuals for whom fixed *f_c_* HSA estimates are most systematically biased. Using measured f_c_ eliminated the significant age effect on errors in females, suggesting that individualized *f_c_* estimation could substantially improve the clinical utility of HSA in this population.

HSA has become a widely used tool in osteoporosis research because it offers geometric information about the proximal femur that standard BMD cannot provide. Beck et al. (29) described how the software derives cross-sectional area, cross-sectional moment of inertia, section modulus, cortical thickness, and buckling ratio from the distribution of mineral mass across pixel profiles at the narrow neck, intertrochanter, and shaft, parameters that reflect how bone resists bending and compressive forces during a fall. Beck et al. (29) acknowledged key limitations: the method reconstructs a three-dimensional structure from a two-dimensional image, is sensitive to leg rotation during scanning, and relies on a fixed assumption about the fraction of mineral mass residing in the cortical compartment. Prevrhal et al. previously compared HSA estimates to volumetric QCT and found reasonable agreement for BMD and CSMI, but acknowledged that no true gold standard existed for the cortical parameters that depend on the *f_c_* assumption (30), leaving this as an open question in the literature that the present study directly addresses.

This limitation is particularly consequential because the cortical parameters most affected by the *f_c_* assumption are also the ones that carry the strongest clinical signal for fracture risk. Kaptoge et al. demonstrated this in what remains the largest prospective validation of HSA to date, following 7,474 women over thirteen years and capturing 635 incident hip fractures (12). Their analysis found that cortical thickness, cross-sectional area, and buckling ratio each predicted hip fracture at least as well as BMD alone at the narrow neck, and that combining geometric variables with age improved discrimination beyond BMD alone. Ha et al. reinforced this from a different angle with a cross-sectional propensity score matched study, finding that hip fracture patients had significantly lower femoral neck cross-sectional area and cortical thickness than matched controls, with HSA variables achieving sensitivities of 82–90% for identifying fracture cases (31). The case for buckling ratio specifically was made most forcefully by Anderson et al., whose twenty-year follow-up of nearly 1,000 Australian women showed that higher shaft buckling ratio remained independently associated with low-trauma fracture even after adjusting for total hip BMD, meaning the information buckling ratio carries about fracture risk is not redundant with BMD and errors in its estimation cannot simply be absorbed by adjusting for BMD in a clinical model (32).

The present findings connect directly to these clinical stakes. The errors associated with fixed *f_c_* documented here are not random but systematic, age-dependent, and sex-specific, meaning they will distort group comparisons across age and sex in precisely the populations where HSA is most commonly applied. The reversal in the direction of BR error between younger and older females is particularly concerning, as younger women are underestimated as stronger than they are while older women are overestimated as weaker than they are, both of which carry implications for clinical decision-making. Replacing fixed *f_c_* with measured *f_c_* significantly reduced errors in *Ct.Ar* for both sexes and in *BR* for males, demonstrating that the *f_c_* assumption is the primary driver of these errors and that they are at least partially correctable. The broader implication is that the HSA parameters most relied upon in clinical fracture risk assessment are also the ones most distorted by an assumption whose empirical basis, as demonstrated above, is uncertain.

The finding that *f_c_* varies systematically with age and *BMC* in females and with weight in males raises the question of whether *f_c_* could be estimated from clinically available parameters and used to improve HSA accuracy without requiring nanoCT imaging. The multivariable regression models explained 36.1% and 40.2% of the variance in *f_c_* for females and males respectively (**Table 3**), and both models were statistically significant. While these *R²* values confirm that the identified predictors capture meaningful variation in *f_c_*, they also indicate that a substantial proportion of the variance remains unexplained, meaning that individual *f_c_* cannot be reliably predicted from age, *BMC*, and weight alone with sufficient precision for clinical application. The sex-specific nature of the predictors adds a further layer of complexity, as a single prediction equation applicable to both sexes is not supported by the data. Nevertheless, these findings demonstrate proof of concept that individualized *f_c_* estimation from clinical measurements is feasible and worth pursuing in larger, more diverse samples. An important next step would be to develop and validate *f_c_* prediction models in a larger cohort with concurrent DXA and QCT or nanoCT data, where the additional structural detail available from volumetric imaging could be used to identify additional predictors and improve model accuracy. Ultimately, a clinically deployable *f_c_* estimation approach, even one that reduces rather than eliminates error, would represent a meaningful improvement over the current fixed assumption and could be implemented within existing HSA software frameworks without requiring additional imaging.

While this study sought to understand how assumptions in HSA calculations lead to systematic error, not every assumption was investigated. The current study was limited to examining errors caused by using a constant *f_c_* versus measured *f_c_* and did not assess methods that estimate CSA directly from the mineral density profile, nor the impact of a non-concentric cortex on HSA parameters. Using 3D nanoCT volumes we found that the narrow neck cross-section is circular but the cortex is not concentric, which is well known (19, 28) and represents an important avenue for future investigation. The current study used a non-Hispanic White donor population spanning a wider age range than the cadaveric samples used in early HSA validation work (15,11. Generalizability across racial and ethnic groups is an important limitation that warrants explicit acknowledgment. Hip fracture rates differ substantially across racial and ethnic groups, with rates approximately 50% lower in African American and Asian women than in White women, and intermediate rates in Hispanic and Native American women (33, 34). Significant differences in femoral neck geometry and composite strength indices have been documented among non-Hispanic White, African American, Mexican American, and Native American women using HSA (35–37), and these structural differences are consistent with the known variation in hip fracture risk across groups. Because the patterns of cortical bone distribution that determine *f_c_* at the narrow neck are likely to differ across racial and ethnic groups in ways that would affect both the magnitude and direction of HSA errors documented here, future studies should characterize *f_c_* in racially and ethnically diverse samples to determine whether population-specific *f_c_* values or correction models are needed for accurate HSA-based fracture risk assessment.

The bone-background and cortical-trabecular FCNNs achieved Dice coefficients of 0.983 ± 0.016 and 0.977 ± 0.023, respectively, in a training cohort of 30 white female donors ranging in age from 36 to 89+ years; Dice was not assessed separately by age group, and future studies should confirm that segmentation accuracy is maintained uniformly across the full age range. Additionally, the training cohort was all-female; whether segmentation performance is equivalent in male specimens, which have a more uniformly distributed cortex and different cortical-trabecular boundary characteristics, has not been formally evaluated. Further, we used pseudoDXA images from nanoCT for actual measurements rather than commercial DXA images, which HSA is generally run on; commercial DXA images of the cadaveric proximal femurs were not available. The discrepancies between fixed and measured *f_c_* are large enough to justify repeating this study on a larger cohort with commercial DXA and nanoCT pseudoDXA for comparison.

Additional limitations warrant acknowledgment. Sample sizes in the male age subgroups were modest (younger: n=18, older: n=12), and *post-hoc* power analyses conducted in R (v 4.6.0, pwr::pwr.t2n.test, *α=0.05*, two-tailed) revealed very low achieved power for the null age-group comparisons of *BR* and *Ct.Ar*: *BR* under fixed *f_c_* (Cohen’s *d=0.20*, *power=8.2%*), *BR* under measured *f_c_* (*d=0.04*, *power=5.1%*), *Ct.Ar* under fixed *f_c_* (*d=0.26*, *power=10.3%*), and *Ct.Ar* under measured *f_c_* (*d=0.26*, *power=10.3%*). These results indicate that the absence of significant age effects in males is most likely attributable to insufficient statistical power rather than a true biological difference from the pattern observed in females, and that a substantially larger male sample would be required to detect age-related differences of the magnitude observed here. The present study computed *f_c_* as the ratio of cortical to total bone voxels at 27 μm resolution, which is a volumetric fraction rather than the mineral mass fraction that the HSA algorithm implicitly requires. Quantitative backscattered electron imaging (qBEI) studies demonstrate that femoral cortical bone exhibits a distinctively higher peak calcium content (*Ca_PEAK_*) than the reference trabecular bone mineral density distribution (*BMDD*) in healthy adults (38–40). Because cortical bone tissue therefore has a higher tissue-level mineral density than trabecular bone, the mineral mass fraction of cortical bone will be systematically higher than its volumetric fraction. Denoting the mean tissue mineral densities of cortical and trabecular bone as *ρ_c_* and *ρ_t_*, and voxel counts as *V_c_* and *V_t_*, the mineral-mass-based *f_c,mass_* that the HSA algorithm requires is:


$$f_{c,mass}=\;\left(\rho_{c}\times V_{c}\right)/\left(\rho_{c}\times V_{c}+\rho_{t}\times V_{t}\right).$$

Since *ρ_c_ > ρ_t_*, *f_c,mass_* exceeds the measured volumetric *f_c,vol_* by a factor between *ρ_c_/ρ_t_*. Lerebours et al. (40) reported mean calcium contents of 25.05 ± 0.28 weight% for femoral cortical bone and 22.20 ± 0.45 weight% for trabecular bone from qBEI measurements, yielding a *ρ_c_/ρ_t_* ratio of approximately 1.13. Applying this correction to mean volumetric *f_c_* values of 0.48 (females) and 0.42 (males) suggests mineral-mass-based *f_c_* values of approximately 0.51 (females) and 0.45 (males), both of which remain well below the assumed 0.6 and do not alter the direction or significance of the sex, age, and weight associations reported here. This systematic upward shift would modestly reduce, but not eliminate, the gap between measured and assumed *f_c_*. Additionally, because the volumetric *f_c_* was substituted into an algorithm expecting *f_c,mass_*, the error reductions reported for the “measured *f_c_*” condition represent a lower bound on the improvement achievable with a true mineral-mass-based measurement. Future studies incorporating voxel-level attenuation calibration to convert volumetric fractions to mineral mass fractions would enable a more complete evaluation of this assumption and may yield further improvements in HSA accuracy. Each nanoCT acquisition included a hydroxyapatite calibration phantom, which in principle could be used to estimate tissue-level mineral density directly from the study’s own calibrated attenuation values, providing an internally derived *ρ*_c_/*ρ_t_* ratio specific to this sample rather than one imported from an independent population. Extraction of per-voxel calibrated attenuation values separately for cortical and trabecular compartments was not feasible within the scope of the present study; this represents a meaningful direction for future work that could replace the externally derived correction factor with a sample-specific estimate. The *f_c_* measured from the 3 mm narrow neck VOI used in the present study is not directly comparable to *f_c_* derived from the 15 mm VOIs used in commercial HSA software, given that *f_c_* varies significantly across VOI locations as shown here, and further study is needed to characterize how VOI-specific *f_c_* differences propagate into HSA algorithm errors. Finally, the weight analyses were conducted within the observed non-obese weight range and may not generalize to individuals with higher body weight.

In conclusion, this study found that the proportion of cortical bone at the narrow neck, represented by *f_c_*, is not a constant 0.6 as used in HSA estimates. *f_c_* differs significantly between females and males and varies with age and pseudoDXA *BMC* in females and with weight in males. Fixed *f_c_* = 0.6 produces large, systematic errors in HSA-derived Ct.Ar and BR that are both sex-specific and age-specific, reaching up to 36% for *Ct.Ar* in both sexes. Using measured *f_c_* substantially reduces errors, with significantly greater improvement in concordance for *Ct.Ar* and *R_inner_* in males. However, systematic errors remained, particularly for *BR* in females, which suggests that a fundamental structural difference, likely the non-uniform cortical thickness distribution, limits the effectiveness of the *f_c_* correction in females and represents the next assumption requiring investigation. In the present study, nanoCT served exclusively as a reference standard for quantifying error and does not constitute a clinically viable pathway for individualized *f_c_* estimation given its radiation dose and limited availability. The practical need arising from this study is the development and validation of models that predict *f_c_* from variables already available on the DXA scan itself, such as attenuation profile, neck geometry, areal density, age, sex, and weight, without requiring additional imaging. The multivariable models presented here, which explained 36.1% and 40.2% of *f_c_* variance in females and males respectively, demonstrate proof of concept for this approach but are insufficient for individual clinical use. These findings motivate further development of DXA-derived *f_c_* prediction models and re-evaluation of HSA-derived fracture risk metrics, particularly in younger women and heavier men.

## Data Availability

The raw data supporting the conclusions of this article will be made available by the authors, without undue reservation.
